# Prevalence and Risk Factors of Neonatal Hyperbilirubinemia in a Semi-Rural Area of the Democratic Republic of Congo: A Cohort Study

**DOI:** 10.4269/ajtmh.23-0293

**Published:** 2023-09-05

**Authors:** Caterina Fanello, Sue Jean Lee, Germana Bancone, Daddy Kayembe, Pauline Ndjowo, Benjamen Badjanga, Gornpan Gornsawun, Paphapisa Chotthanawathit, Naomi Waithira, Nicholas John White, Marie Onyamboko

**Affiliations:** ^1^ Nuffield Department of Medicine, Centre for Tropical Medicine and Global Health, University of Oxford, Oxford, United Kingdom;; ^2^ Mahidol-Oxford Tropical Medicine Research Unit, Faculty of Tropical Medicine, Mahidol University, Bangkok, Thailand;; ^3^ Kinshasa-Oxford Medical Research Unit, Kinshasa, Democratic Republic of Congo;; ^4^ Shoklo Malaria Research Unit, Mahidol-Oxford Tropical Medicine Research Unit, Faculty of Tropical Medicine, Mahidol University, Mae Sot, Thailand;; ^5^ Kinshasa School of Public Health, University of Kinshasa, Kinshasa, Democratic Republic of Congo

## Abstract

Neonatal hyperbilirubinemia (NH) is a frequent condition that, if left untreated, can lead to neurological disability and death. We assessed the prevalence of NH and associated neonatal and maternal risk factors in 362 mothers and 365 newborns in a semi-rural area of the Democratic Republic of Congo. In addition, we explored the knowledge and practices of mothers regarding this condition. We collected demographic data, anthropometric data, and obstetric and medical anamneses. We examined newborns at birth and at 24, 48, and 72 hours and measured bilirubin at birth in umbilical cord and capillary blood and thereafter in capillary blood. Hemoglobin, hematocrit, ABO group, Rhesus factor, glucose-6-phosphate dehydrogenase (G6PD) deficiency, Hemoglobin S (HbS), and malaria were assessed in mothers and newborns. Among 296 newborns (all time points available), 5.7% developed NH (95% CI: 3.4–9.0) between 24 and 72 hours according to National Institute for Health and Care Excellence (NICE) UK guidelines. There was a significantly higher risk in newborns with G6PD deficiency (homo- and hemizygous adjusted Odd Ratio [aOR]: 21.0, 95% CI: 4.1–105.9), preterm births (aOR: 6.1, 95% CI: 1.4–26.9), newborns with excessive birth weight loss (aOR: 5.8, 95% CI: 1.4–23.2), and hyperbilirubinemia at birth (aOR: 14.8, 95% CI: 2.7–79.6). Newborns with feto-maternal ABO incompatibility and G6PD deficiency had significantly higher bilirubin at birth than others. More than 60% of mothers had adequate knowledge of NH, but compliance with phototherapy in the absence of symptoms was low. Although risk factors for NH are common in this area, prevalence was not high, suggesting a need for better case definition. Implementation of point-of-care devices for diagnosis and awareness programs on risk prevention could help reduce neonatal morbidity and mortality associated with hyperbilirubinemia in these areas.

## INTRODUCTION

Neonatal hyperbilirubinemia (NH) is common among healthy newborns and usually resolves spontaneously within a week. The excess of unconjugated bilirubin in plasma is the result of a transient imbalance between bilirubin production, mostly derived from the breakdown of red blood cells, which have a higher concentration and a shorter life span than in adults, and its elimination by the immature liver. Conditions that increase bilirubin production and/or decrease its excretion may exacerbate this imbalance. Unconjugated bilirubin is a neurotoxic lipid-soluble compound that, if in excess, can cross the blood-brain barrier causing acute and chronic encephalopathy and long-term neurological sequelae (kernicterus spectrum disorder) and can lead to neonatal death. Morbidity and mortality are largely preventable if cases are identified and treated early. Where available, effective treatment includes blue-light phototherapy and in severe cases exchange transfusion. Known neonatal and maternal factors associated with an increased risk of NH are preterm birth (< 37 weeks of gestational age), low birth weight (LBW; < 2,500 g) or small for gestational age (SGA), maternal-fetal ABO or Rhesus incompatibility, and inherited neonatal disorders such as glucose-6-phosphate dehydrogenase (G6PD) deficiency and Gilbert’s syndrome. Postnatal factors include birth trauma, infections, inadequate breastfeeding and dehydration in the first few days of life, and exclusive breastfeeding (late-onset hyperbilirubinemia). Pregnancy-associated complications and intercurrent diseases that increase the risk of intrauterine growth restriction (IUGR), preterm birth, or LBW/SGA can increase the risk of NH.

It is estimated that worldwide this condition affects at least 481,000 term or near-term newborns annually, causing 114,000 deaths and more than 63,000 cases of moderate or severe disability.^1^ In high-income settings, early diagnosis and treatment have dramatically improved outcomes for newborns at risk. However, in low- and middle-income countries, true population-based data are scarce, especially from rural areas, and the contribution of hyperbilirubinemia to neonatal morbidity and mortality remains unclear. A recent meta-analysis of data from 12,327 infants in four sub-Saharan African countries estimated that more than one in four babies had hyperbilirubinemia (28%; 95% CI: 20–36%) with large differences across and between countries (ranging from 4.9% to 44.9%).^2^ These differences are probably caused in part by the use of different methods of diagnosis and different case definitions applied.

The Democratic Republic of Congo (DRC) has one of the highest neonatal mortality rates in the world: 27 per 1,000 live births.^3^ Little is known about the risk of NH and the extent of its contribution to morbidity and mortality. Diagnosis is usually made by visual inspection, which has low accuracy,^4^^–^^6^ and it is not systematically reported in maternity records.^7^ Risk factors for NH are common. It is estimated that 12–18% of newborns are preterm and 10% are LBW,^8^^,^^9^ there is a high prevalence of G6PD deficiency,^10^^,^^11^ and hemoglobin S (HbS) (sickle cell trait and sickle cell disease [SCD]),^12^ and malaria is endemic in the country. Malaria in pregnancy increases the risk of IUGR, preterm birth, and LBW and SGA newborns. Likewise, those born to women with SCD are at greater risk of being preterm or LBW/SGA, with hyperbilirubinemia being a common complication.^13^ Furthermore, studies conducted in similar settings (e.g., Nigeria and Ghana) on knowledge, attitudes, and practices toward NH among expectant mothers and caregivers have shown that limited knowledge and poor attitude can lead to delays in seeking care as well as delays in treatment.^14^^–^^17^ In this study, we evaluated the prevalence of NH in a cohort of in-hospital births in a semi-rural area of Kinshasa (DRC) and the association between NH and neonatal and maternal risk factors. We also investigated expectant mothers’ knowledge and practices regarding this condition.

## MATERIALS AND METHODS

### Study design and setting.

This prospective observational study was conducted at the antenatal care (ANC) center and maternity ward of Maluku Referral Hospital, Kinshasa, DRC. The hospital is located in a semi-rural area and serves an estimated population of ∼200,000 people. Women were invited to participate when they attended their last ANC visit or on the day of expected delivery and were enrolled on the day of delivery. We included mothers of any age who consented to participate and to stay 72 hours in the hospital after giving birth. We excluded newborns whose health conditions in the judgment of the clinician made it difficult to comply with study procedures. Participants were invited to come back with their newborn for a follow-up visit at day 7.

This study was conducted in agreement with the Declaration of Helsinki ethical principles for medical research involving human subjects. Research ethics approval was given by the research ethics committee of the Kinshasa School of Public Health (ESPI/CE/013/2019) and the University of Oxford (OxTREC, 505-19). The study was approved by the Ministry of Health of the DRC. All participants agreed in writing before enrollment and after receiving adequate explanation in their preferred language. For women who agreed to participate at the ANC visit, full informed consent was obtained on that day, and a signed confirmation was sought on the day of delivery (two-step informed consent). The aim was to interfere as little as possible with hospital procedures and to avoid further stress to the mother during labor. Women who were invited the day of delivery were consented before childbirth.

### Mother data collection.

Demographic and anthropometric data, self-reported obstetrical, medical, and drug history in the month prior to enrollment, were collected. Young maternal age (teenager) was defined as < 19 years old.^18^ Gestational age (EGA) was estimated from date of the last menstrual period (LMP). If the mother remembered only the month, we considered the 15th as the LMP date, and if she did not remember the month, we used the fundal height (available from ANC center records). Vital signs were measured, and a venous blood sample was taken to measure hemoglobin (Hb) and hematocrit (HCT), determine the ABO blood group and Rhesus factor, and diagnose malaria and G6PD deficiency. Clinical signs of suspected infections were reported. Qualitative data were generated from semi-structured interviews with the mothers who participated in the study using a validated questionnaire.^19^

### Newborn data collection.

Newborns were examined immediately after birth. Data on sex, weight, length, head circumference, vital signs, and Apgar scores at 1, 5, and 10 minutes of life were collected. Newborns were evaluated for visible signs of hyperbilirubinemia. A blood sample was taken from umbilical cord blood (UCB) and heel prick (capillary blood). Total serum bilirubin (TSB) and direct bilirubin (DSB) levels were measured at birth from UCB and capillary blood and at 24, 48, and 72 hours or until the start of phototherapy from capillary blood. In a subgroup, total serum bilirubin (TSB) and direct serum bilirubin (DSB) were measured also at day 7. The UCB sample was used to measure Hb and HCT and diagnose malaria and G6PD deficiency. Preterm birth was defined as occurring before 37 weeks, LBW as less than 2,500 g, and SGA and large for gestational age (LGA) as less than the 10th centile and higher than the 90th centile, respectively, using INTERGROWTH-21 standards.^20^ Excessive neonatal weight loss was defined as a loss of more than 7% of weight within 72 hours^21^ or until NH diagnosis, whichever came earlier. ABO incompatibility was defined as the mother having blood group O and the newborn having A or B, and Rhesus incompatibility when a mother was Rh− with a newborn Rh+. Newborns who needed phototherapy were transferred to the neonatal intensive care unit of Kingasani Maternity Hospital. Sun exposure was used if the mother refused phototherapy and medical counseling failed. In this case, mothers were asked to expose their babies to sunlight daily for about 45 minutes early in the morning (usually from 8:00 to 8:45 a.m.) for 3 to 5 days at the hospital and back home. Maternal and neonatal infection diagnosed during the course of the study was treated according to DRC National Guidelines.

### Laboratory methods.

Bilirubin was measured using a FUJI DRI-CHEM 4000i automated system. Malaria was diagnosed by Rapid Diagnostic Test (RDT; CareStart™ Malaria, Access Bio, Somerset, NJ) and standard microscopy methodology. In a subgroup of newborns, malaria was assessed using ultrasensitive RDTs (Alere *Malaria* Ag P.f., Abbott, Chicago, IL). Hemoglobin and HCT were measured by HemoCue^®^ 301 (Angelholm, Sweden) and Hawksley Haematospin 1400 (Hawksley & Sons, Ltd., Lancing, United Kingdom), respectively. Anemia in the mother was defined as mild, moderate, or severe using WHO cutoffs.^22^ Polycythemia in the newborn was defined as venous HCT ≥ 65% or Hb ≥ 22 g/dL.^23^ The ABO group and Rhesus type were assessed by the Beth-Vincent and Coombs tests, respectively. Glucose-6-phosphate dehydrogenase deficiency was assessed by RDT (CareStart™ G6PD Access Bio) and Fluorescent Spots Test (FST; R&D Diagnostic Ltd., Papagos, Greece). A dried blood spot was collected from the mother and her newborn(s) for identification of HbS (rs334) and G6PD 202G>A (rs1050828) from genomic DNA, using published DNA extraction^24^ and the polymerase chain reaction - restriction fragment length polymorphism (PCR-RFLP) methods.^25^^,^^26^

### Statistical analysis.

The primary outcome was the prevalence of NH at 24, 48, and 72 hours, defined as a TSB level above the threshold for phototherapy according to National Institute for Health and Care Excellence (NICE) nomograms based on EGA at birth.^27^ Sample size calculations were based on the number of births occurring annually at the maternity ward of Maluku Referral Hospital. A sample size of 306 newborns allowed the detection of a 15% prevalence with ±4% precision. This sample size also allowed for the detection of higher prevalence with decreasing precision (prevalence 45%, ±6%) or lower prevalence with increased precision (prevalence 5%, ±2.5%). Mothers who left the maternity ward before 72 hours were replaced. We analyzed the data of newborns who were diagnosed with NH or for whom data were available up to 72 hours. Maternal and neonatal factors were described using means (SD), medians (interquartile range [IQR]), and frequencies (percentage, %). Maternal and neonatal risk factors were compared using the Student’s *t* test, Mann-Whitney test, Wilcoxon matched-pairs sign rank test, or χ^2^ test, as appropriate. The average rate of change of TSB over time was estimated by calculating slopes for each newborn. To identify predictors of NH, variables with a *P* value < 0.02 from the univariate analysis were included in a multivariable logistic regression model along with known risk factors: young maternal age, preterm birth, LBW, SGA, maternal-fetal ABO and Rhesus incompatibility, G6PD deficiency, malaria and other infections, excessive neonatal weight loss, and maternal SCD. Using a stepwise approach, only factors that were significant at *P* < 0.05 were retained in the final model. Twins were excluded from the risk factor analysis because of violation of the assumption of independence of data. Similarly, to avoid collinearity, SGA was considered without LBW and preterm birth (and LBW and preterm birth were considered without SGA) in the separate models and were assessed using Akaike’s information criterion and log likelihood. Breastfeeding was not included as a risk factor because all mothers breastfed their babies. All models were adjusted for bilirubin levels at birth. A cutoff for the prediction of NH using UCB was identified using area under the operating characteristic curves and the Youden index. Quantitative and qualitative data were entered using MACRO and analyzed with STATA version 17.0 (StataCorp LLC, College Station, TX).

## RESULTS

**Figure 1.**
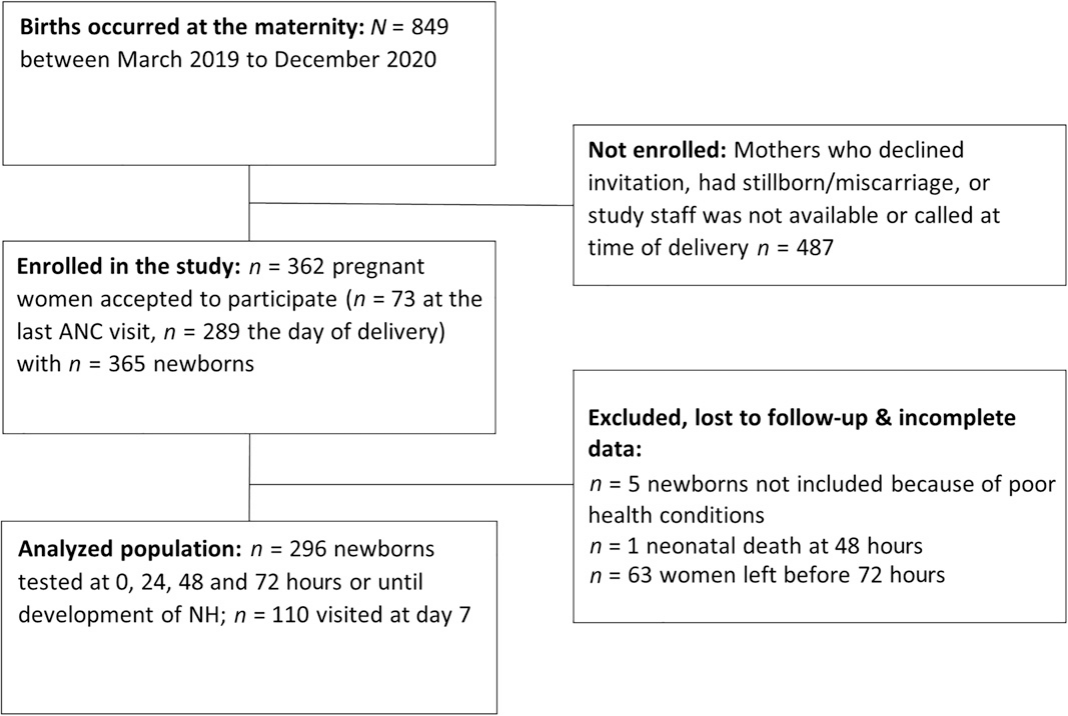
Flowchart. ANC = antenatal care; NH = neonatal hyperbilirubinemia.

Between March 2019 and December 2020, 849 births occurred at Maluku Hospital. In total, 362 mothers with 365 newborns were enrolled (Figure 1). Adherence to protocol was low, and 63 mothers left the maternity ward before 72 hours. One in-hospital newborn death occurred at 48 hours for unascertained causes. Overall, data at all planned time points were collected for 296 newborns. About a third of the mothers (*N* = 110) came back for a visit after 1 week, and their newborns were visited and tested for bilirubin.

### Mother cohort (data summarized in Table 1).

**Table 1:** Maternal anthropometric, demographic, clinical, and laboratory results

Characteristics	Summary measure
Enrolled, *n*	362*
Agreed to participate at last ANC visit	73 (20.2%)
Unable to recall LMP date	46 (12.7%)
Median maternal age (IQR), *n* = 360	25.3 (20.8, 32.1)
Teenager, *n* (%)	49 (13.6%)
Mean maternal weight at birth, kg (SD)	64.3 (9.8)
Mean maternal height, cm (SD)	162.8 (6.4)
Obstetric history	
Primigravid, *n* (%)	100 (27.6%)
Median gravidity (IQR)	2 (1–4)
Previous preterm pregnancy†	2 (0.76%)
Previous newborn with NH†	4 (1.53%)
Medical history (for the current pregnancy)	
Hypertensive disorder, *n* (%)	2 (0.6%)
Maternal diabetes, *n* (%)	1 (0.3%)
Adhered to IPTp-SP, *n* (%)	341 (94.2%)
Median number of SP doses (IQR)	3.0 (2.0–3.0)
Maternal infection in previous month	46 (12.7%)
Prescribed antibiotics, *n* (%)	45/46 (97.8%)
Data at delivery	
Median gestational age at delivery, weeks (IQR)	39.4 (38.0–40.6)
Mean hematocrit, % (SD)	33.9 (4.4)
Mean hemoglobin, g/dL (SD)	11.3 (1.5)
Anemia, *n* (%)	
Normal (11.0+ g/dL)	228 (63.0%)
Mild (10.0–10.9 g/dL)	77 (21.3%)
Moderate (7.0–9.9 g/dL)	56 (15.5%)
Severe (< 7.0 g/dL)	1 (0.3%)
Median body temperature, °C	36.5 (36.2–36.8)
Suspected maternal sepsis, *n* (%)	1 (0.3%)
*Plasmodium falciparum* (RDT), *n* (%)	61 (16.9%)
*P. falciparum* (microscopy), *n* (%)	60 (16.6%)
Geo. mean parasitemia/µL (95% CI)	1,341 (696–2,586)
Range	32–195,000
Genetic data	
Sickle cell trait, *n* (%)	75/356 (21.1%)
Sickle cell disease, *n* (%)	1/356 (0.3%)
G6PD deficiency RDT, *n* (%)	5/226 (2.2%)
G6PD deficiency FST, *n* (%)	7/360 (1.9%)

ANC = antenatal care; FST = Fluorescent Spots Test; Geo. = Geometric; G6PD = glucose-6-phosphate dehydrogenase; IPTp-SP = intermittent preventive treatment with sulphadoxine-pyrimethamine; IQR = interquartile range; LMP = last menstrual period; NH = neonatal hyperbilirubinemia; RDT = Rapid Diagnostic Test.

* Total analyzed if not otherwise indicated.

† Percentage calculated from multiparous women.

Most women (79.8%) agreed to participate on the day of delivery, and 20.2% agreed at their last antenatal appointment. The median age was 25.3 years (IQR: 20.8–32.1), and 13.6% were teenagers. The majority of mothers (72.4%) were multigravidae (median number of pregnancies, *N* = 2; IQR: 1–4). The most frequently reported medical condition was urinary and/or genital tract infection (12.7%). Two women reported having been diagnosed with hypertensive disorder and one with maternal diabetes. Only two women reported having had a previous preterm pregnancy, and four had a previous newborn with clinical jaundice. Most women (94.2%) reported having adhered to the malaria intermittent preventive treatment with sulphadoxine-pyrimethamine (median number of doses *N* = 3; IQR: 2–3). Median EGA at delivery was 39.4 weeks (IQR: 38.0–40.6). Ten women could not recall their LMP date, and 36 provided only the month. This group of women was younger than the entire cohort: median age 22 years (IQR: 19.4–26.1 years) versus 25.9 years (IQR: 20.9–32.7 years); *P =* 0.003. Mean (SD) Hb was 11.3 (1.5) g/dL, with 37% of women having mild to severe anemia. Sixty-one women (16.9%) were positive for malaria by RDT, and 60 were confirmed by microscopy as having *Plasmodium falciparum* mono-infection, with a mean parasitemia of 1,341 trophozoites/µL (95% CI: 696–2,586). Malaria occurred more frequently in primigravidae (30.0% versus 11.8%, *P <* 0.001) and in younger women (median age, 22.7 years; IQR: 19.5–27.0 years versus 26.0 years; IQR: 20.9–32.9 years; *P =* 0.005). The allelic frequency of HbS was 10.8% (75 heterozygotes and 1 homozygote, out of 356 women). The prevalence of G6PD deficiency was 1.9% by FST and 2.2% by RDT.

### Newborn cohort (data summarized in Table 2).

**Table 2:** Neonatal anthropometric, demographic, clinical, and laboratory results

Characteristics	Summary measure
Enrolled, *n*	365*
Male, *n* (%)	190 (52.1%)
Twins, *n* (%)	11 (3.0%)
Mean height, cm (SD)	49.3 (2.3)
Mean head circumference, cm (SD)	34.3 (1.5)
Mean birth weight, g (SD)	3,121.0 (456.8)
Low birth weight (< 2,500 g), *n* (%)	25 (6.8%)
Preterm birth, *n* (%)	53 (14.5%)
Small for gestational age, *n* (%)	67/357 (18.8%)
Large for gestational age, *n* (%)	44/357 (12.3%)
Mean temperature, °C (SD)	36.2 (0.5)
Apgar score < 7 at 1 minute, *n* (%)	27 (7.4%)
Apgar score < 7 at 5 minutes, *n* (%)	5 (1.4%)
Apgar score < 7 at 10 minutes, *n* (%)	0
Mean pulse (SD), beats/minute	143.1 (9.9)
Mean respiratory rate, breaths/minute (SD)	54.0 (4.8)
Mean hematocrit, % (SD)	46.2 (5.0)
Mean hemoglobin, g/dL (SD)	15.3 (1.6)
Suspected sepsis, *n* (%)	3 (0.8%)
*Plasmodium falciparum* (RDT), *n* (%)	2 (0.6%)
*P. falciparum* (usRDT), *n* (%)	3/120 (2.5%)
*P. falciparum* (microscopy), *n* (%)	1 (0.3%)
ABO incompatibility, *n* (%)	46/364 (12.6%)
Rhesus incompatibility, *n* (%)	4 (1.1%)
Loss of > 7% birth weight, *n* (%)	39/299 (13.0%)

RDT = Rapid Diagnostic Test; usRDT = ultrasensitive rapid diagnostic test.

* Total analyzed if not otherwise indicated.

All 365 births occurred by vaginal delivery. Of these, 11 were twins (in three cases, both siblings were included and in five cases only one). Mean (SD) birth weight was 3,121 (456.8) g, and 6.8% were LBW. Fifty-three newborns (14.5%) were preterm, 18.8% SGA, and 12.3% LGA. The mean Apgar scores at 1, 5, and 10 minutes were 8.4, 9.2, and 9.9, respectively; 7.4% had a score < 7 at 1 minute and 1.4% at 5 minutes, and none had a decrease between 5 and 10 minutes. None of the newborns presented with discoloration of the bulbar conjunctiva, skin, palm, or sole. Three newborns had clinically suspected sepsis (0.8%). Forty-six dyads (12.6%) had feto-maternal ABO incompatibility, and six (1.7%) had Rhesus incompatibility (but only four mothers were multigravidae). None of the newborns had polycythemia. All mothers breastfed, with most starting immediately (99.5%). Within the first 72 hours (or until diagnosis of NH), 39 newborns lost ≥ 7% of their birth weight. The UCB was positive for malaria in three newborns by ultra sensitive RDT (usRTD), two by standard RDT, and one by microscopy (64 trophozoites/µL, *P. falciparum* mono-infection, not identified by RDT). The mothers of these five newborns also had malaria. The allelic frequency of G6PD A- (202G>A) was 14.6% in males and 15.5% in females (Table 3). Glucose-6-phosphate dehydrogenase deficiency prevalence by FST was 3.3%. When compared with PCR results (results available for both tests, *N* = 348), FST detected 26.9% of the hemizygous males (7/26) and 33.3% of the homozygous females (1/3). The prevalence by RDT was 3.1% and, compared with PCR results (results available for both tests, *N* = 213), detected 25% of the hemizygous males (4/16) and the only homozygous female. Both FST and RDT failed to detect any of the heterozygous females.

**Table 3:** G6PD deficiency in newborns

Test	Overall	Females	Males
*n*	365	175	190
FST G6PD			
Normal	352/364 (96.7%)	172/174 (98.9%)	180/190 (94.7%)
Deficient	12/364 (3.3%)	2/174 (1.1%)	10/190 (5.3%)
RDT G6PD			
Normal	217/224 (96.9%)	108/109 (99.1%)	109/115 (94.8%)
Deficient	7/224 (3.1%)	1/109 (0.9%)	6/115 (5.2%)
PCR			
Wild type	273/349 (78.2%)	121/171 (70.8%)	152/178 (85.4%)
Heterozygous	47/349 (13.5%)	47/171 (27.5%)	0/178 (0.0%)
Hemi- or homozygous	29/349 (8.3%)	3/171 (1.8%)	26/178 (14.6%)

FST = Fluorescent Spots Test; G6PD = glucose-6-phosphate dehydrogenase; PCR = polymerase chain reaction; RDT = Rapid Diagnostic Test.

### Neonatal hyperbilirubinemia.

**Figure 2.**
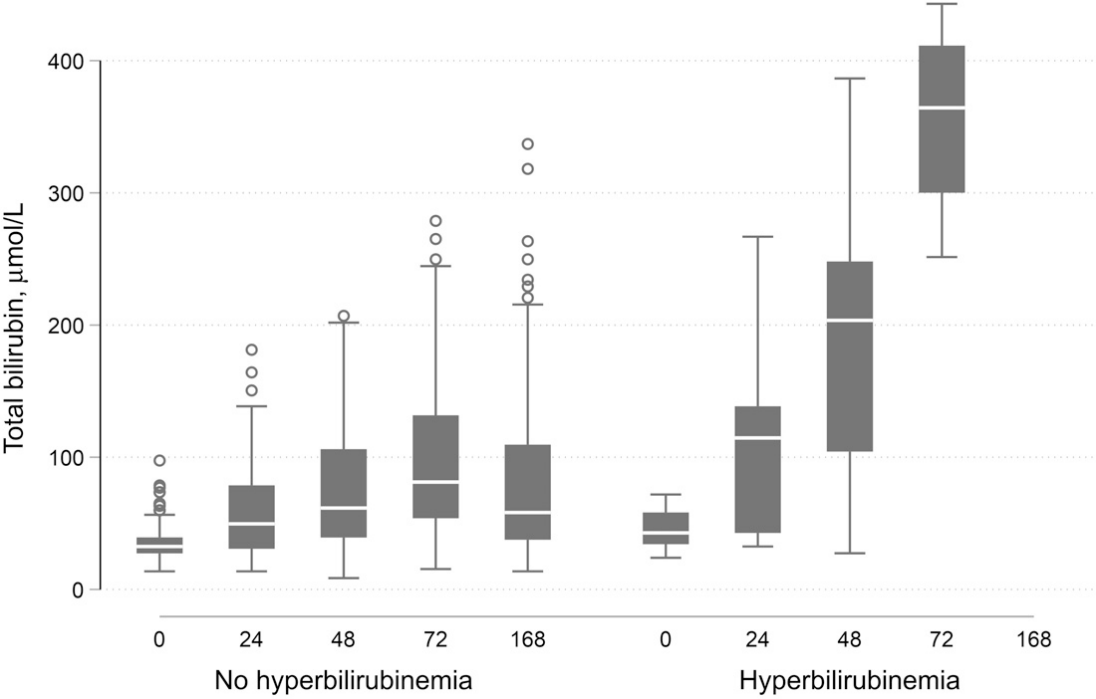
Median interquartile range total bilirubin level over time in newborns without and with neonatal hyperbilirubinemia.

**Figure 3.**
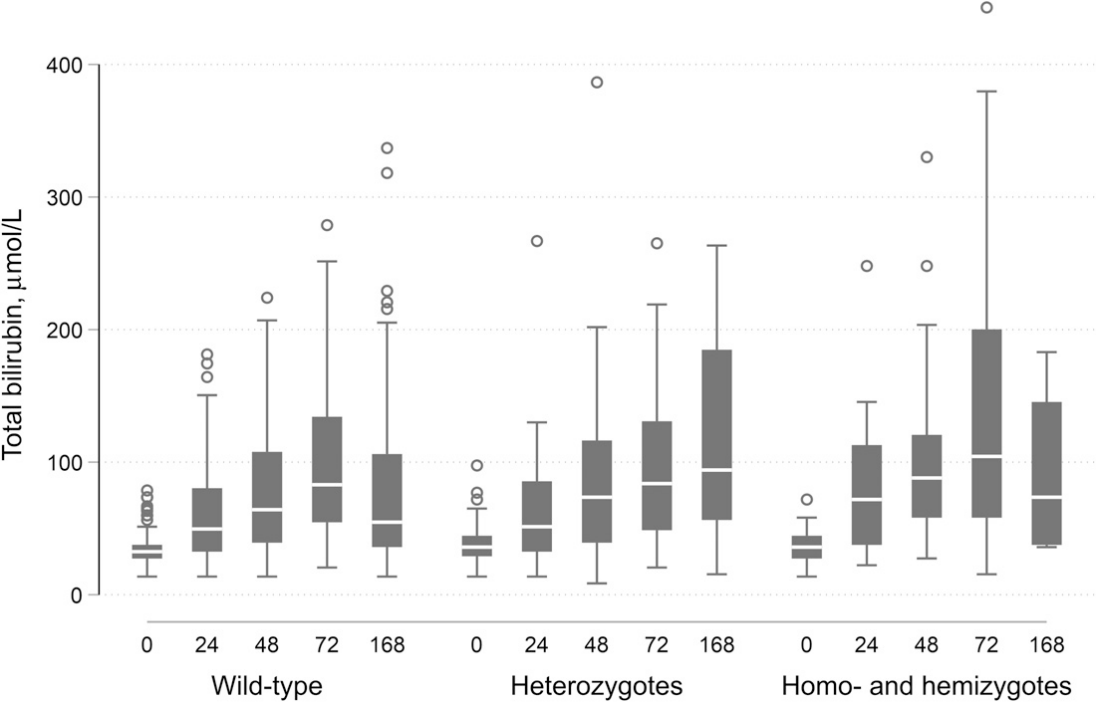
Median interquartile range total bilirubin level over time by glucose-6-phosphate dehydrogenase genotype.

At birth, bilirubin was measured in both UCB and capillary blood in all 365 newborns. In 19 cases, the value in capillary blood was higher than 100 µmol/L (for term newborns) or 40 µmol/L (for preterm newborns). These cases were kept under observation, and bilirubin was measured again at scheduled time points, except for one case whose mother self-discharged at 48 hours. Median (IQR) TSB in capillary blood was significantly higher than in cord blood: 34.2 (27.4–39.3) µmol/L versus 27.4 (22.2–35.9) µmol/L; *P <* 0.001 (*N* = 365). Total serum bilirubin was also significantly higher among newborns with ABO incompatibility (*P =* 0.035 for capillary blood and *P =* 0.031 for UCB) and in those with G6PD deficiency (homo- and hemizygous; *P =* 0.022 for capillary blood and *P <* 0.001 for UCB; Table 4). After birth, TSB measurements were available at all time points (24, 48, and 72 hours or until NH diagnosis) for 296 newborns. On average, TSB levels increased from birth in 85.5% of cases (positive slope in 253/296) and rose above the threshold for phototherapy in 17 newborns (5.7%): eight cases at 24 hours (none above 17 mg/dL), five cases at 48 hours (two above 17 mg/dL), and four cases at 72 hours (three above 17 mg/dL). Newborns who were diagnosed with NH between 24 and 72 hours had a median TSB increase of 4.13 μmol/L per day (IQR: 3.49–5.35) compared with 0.51 μmol/L (IQR: 0.13–1.18) in those without NH (*P* < 0.001; Figure 2). Newborns homozygous or hemizygous for G6PD deficiency had on average a median increase in bilirubin of 0.87 μmol/L per day (IQR: 0.15–3.53 μmol/L), which was not different from wild types (0.61 μmol/L; IQR: 0.15–1.31 μmol/L) or heterozygotes (0.45 μmol/L; IQR: 0.12–1.61 μmol/L; *P* = 0.633; Figure 3). In the group of newborns who were brought to the hospital for a follow-up visit between days 6 and 8, one new case was identified and one case was readmitted with clinical jaundice (the latter was diagnosed at 24 hours, but the parents refused treatment and left the hospital). Among cases that qualified for phototherapy treatment, only five mothers agreed to transfer their newborn to the neonatal intensive care unit (ICU; 29.4%). Some mothers accepted sunlight exposure in lieu of transfer to the ICU.

**Table 4:** Median (IQR) bilirubin levels (TSB, µmol/L)

Characteristics	*n*	0 Hours (capillary)	*n*	0 Hours (UCB)	*n*	24 Hours	*n*	48 Hours	*n*	72 Hours
Neonatal hyperbilirubinemia										
No	279	32.5 (27.4–39.3)	279	27.4 (22.2–34.2)	279	49.6 (30.8–80.4)	279	54.7 (35.9–100.9)	279	68.4 (42.8–124.9)
Yes	17	42.8 (34.2–58.2)	17	35.9 (29.1–41.0)	17	114.6 (42.8–138.5)	15	147.1 (94.1–224.1)	12	227.5 (108.6–364.3)
ABO incompatible*										
No	258	32.5 (27.4–39.3)	258	27.4 (22.2–34.2)	258	49.6 (30.8–80.4)	256	54.7 (35.9–104.3)	253	70.1 (42.8–128.3)
Yes	37	35.9 (30.8–46.2)	37	32.5 (23.9–41.0)	37	49.6 (32.5–92.4)	37	70.1 (53.0–106.0)	37	71.8 (42.8–145.4)
G6PD†										
Wild type	221	32.5 (27.4–39.3)	221	27.4 (22.2–34.2)	221	49.6 (30.8–82.1)	221	58.2 (35.9–106.0)	220	71.8 (44.5–129.1)
Heterozygous	40	35.9 (29.1–50.5)	40	29.9 (24.8–41.9)	40	50.5 (30.8–84.7)	39	61.6 (37.6–106.0)	39	78.7 (44.5–130.0)
Hemi- or homozygous	21	39.3 (30.8–51.3)	21	35.9 (34.2–41.0)	21	71.8 (37.6–97.5)	20	77.0 (39.3–108.6)	19	87.2 (27.4–200.1)
Preterm birth										
No	256	32.5 (27.4–40.2)	256	27.4 (22.2–35.9)	256	49.6 (30.8–83.8)	254	56.4 (35.9–106.0)	251	71.8 (42.8–130.0)
Yes	40	32.5 (28.2–42.8)	40	28.2 (23.9–37.6)	40	47.9 (30.8–75.3)	40	59.9 (41.0–102.6)	40	68.4 (41.9–136.8)
Loss of > 7% birth weight										
No	255	32.5 (27.3–39.3)	260	27.4 (22.2–35.9)	255	49.6 (30.8–82.1)	253	59.8 (35.9–104.3)	251	71.8 (42.8–130.0)
Yes	36	35.0 (26.5–43.7)	36	29.1 (22.2–36.8)	36	51.3 (34.2–87.2)	36	55.6 (33.4–99.2)	35	75.2 (54.7–157.3)

G6PD = glucose-6-phosphate dehydrogenase; IQR = interquartile range; TSB = total serum bilirubin; UCB = umbilical cord blood.

* *P* = 0.035 for comparison of 0 hours (capillary), and *P* = 0.031 for comparison of 0 hours (UCB).

† *P* = 0.022 for comparison of 0 hours (capillary), and *P* < 0.001 for comparison of 0 hours (UCB).

### Risk factors for NH.

The neonatal risk factors associated with NH were G6PD deficiency (independent of the method used for diagnosis; *P <* 0.01), preterm birth (*P =* 0.007), excessive loss of birth weight in the first 72 hours (*P =* 0.025), and suspected neonatal sepsis (*P <* 0.001; Supplemental Table 1). There was a borderline association with higher mean Hb levels at birth (*P =* 0.045) and a lower median EGA at birth (*P =* 0.046). The only maternal risk factor significantly associated with NH was G6PD deficiency diagnosed by FST (*P =* 0.009; Supplemental Table 2). Multivariable analysis identified newborns with G6PD deficiency being 21 times more likely to develop NH compared with wild-type newborns (homozygous females and hemizygous males, adjusted Odd Ratio (aOR): 21.0; 95% CI: 4.1–105.9; heterozygous females: aOR: 9.1; 95% CI: 1.5–53.5). The odds of NH were also higher among preterm births (aOR: 6.1; 95% CI: 1.4–26.9) and newborns with excessive birth weight loss (aOR: 5.8; 95% CI: 1.4–23.2). The risk of NH increased with increasing levels of bilirubin at birth (aOR: 14.8; 95% CI: 2.7–79.6).

### Predictive value of UCB bilirubin for postnatal hyperbilirubinemia.

Using TSB from UCB, the cutoff identified by the Youden index to predict NH was 25.7 μmol/L, which gave a sensitivity of 100% (95% CI: 85–100%), a specificity of 36.2% (95% CI: 30.6–42.1%), and an area under the receiver operating characteristic (ROC) curve of 68.1 (95% CI: 65.3–70.9). However, as the maximum calculated Youden index was below 50% (36.2%), this indicates that UCB screening would not be a useful test in the study population. Noting that the positive and negative predictive values (PPV and NPV, respectively) are dependent on prevalence, the PPV was 8.72% (95% CI: 5.16–13.6%) and the NPV was 100% (95% CI: 96.4–100%).

## KNOWLEDGE AND PRACTICE OF NEONATAL JAUNDICE IN EXPECTANT MOTHERS

Most women (98%) agreed to complete the questionnaire (data are summarized in Supplemental Table 3). About half (52%) had a secondary or higher level of education, 45% had a primary level education, and 3% had no education. Only 3.7% were employed. Participants belonged to 78 different ethnic groups, reflecting the large ethnic variability of the DRC (> 250 ethnic groups). The most represented (each ≥ 5% of the sample) belonged to the main cultural clusters of the center-south of the country.

### Knowledge of jaundice.

More than half of the women (65.5%) had heard of jaundice before the survey and were able to recognize a jaundiced newborn when shown a picture (59.6%). Women who were older and/or multiparous and had a higher level of education were significantly more likely to have heard of jaundice and to be able to identify a jaundiced newborn from a picture (*P =* 0.016 and *P* < 0.001, respectively). Nurses and community health workers were the most frequently reported source of information (84.5%). Four women (1.1%) reported that they had a previous newborn with jaundice, and none received phototherapy (one sunlight exposure, one sweetened water, one oral traditional treatment, and one an unspecified drug).

### Knowledge of causes, symptoms, and consequences.

Just over 1 in 10 women (11.9%) reported that they knew why a newborn could become jaundiced, and most attributed it to an infection or illness of the newborn and/or of the mother. Regarding symptoms, 59.9% of women felt that they were able to recognize them, and 61.3% correctly identified a yellowish discoloration of the skin and eyes as a sign, which was considered harmful by most women (59.3%). Regarding complications, 69.8% of respondents agreed that if a newborn becomes very jaundiced, he/she might become sick, might refuse to feed (47.1%), might develop convulsions (21.2%), might develop brain damage (29.7%), and might die (61.3%), whereas 22% of respondents did not know of any complications that can result from it. Regarding long-term consequences, 58.5% of respondents agreed that jaundice could result in death, delayed development (30.5%), brain damage (29.1%) and convulsions (21.8%), or deafness (10.5%).

### Practice.

The majority (98.6%) of mothers reported that they would bring their newborn to the hospital if they developed the symptoms of jaundice; 6.8% would use an herbal/traditional treatment either orally or by topical application, or would give more milk (1.4%), and 3.1% would expose the newborn to sunlight. A minority (11%) used naphthalene (mothballs) in the house and for storing newborn’s clothes (5%); 2.3% used menthol or camphor-based ointments on their skin. Most women (96.9%) stated that they would be interested in participating in a focus group to discuss this problem in the community, and 96.6% were happy to be contacted in the future for this purpose (data not shown).

## DISCUSSION

In this cohort of newborns in a semi-rural area of Kinshasa, hyperbilirubinemia occurred in 6% of cases in the first 3 days of life. As observed elsewhere, G6PD-deficient newborns had the highest risk of developing hyperbilirubinemia owing to a mechanism only partially explained by increased hemolysis.^28^^,^^29^ Glucose-6-phosphate dehydrogenase deficiency is frequent in Africa (mostly caused by 202G>A mutation) and is common in this area.^11^ Early screening for this condition would help to assess which newborns are at risk of developing severe hyperbilirubinemia before they are discharged from the hospital, but diagnosis in resource-limited settings remains challenging. Rapid lateral flow diagnostic tests and the fluorescent spot technique are easy to use and accessible but identify only individuals with low enzyme activity (< 30%); thus, they are unable to diagnose most heterozygous females with intermediate enzyme activity.^30^ Sensitivity further decreases in newborns because of the high number of circulating reticulocytes, which have a higher enzyme expression than mature red blood cells (RBCs).^31^ In our study, both RDT and FST tests missed all heterozygous cases and a substantial percentage of hemizygous and homozygous newborns. A novel quantitative point of care (POC) device has been used in cord blood with a higher sensitivity and good usability by clinical workers in resource-limited settings^32^ and is a viable practical approach that could be considered in the DRC.

Preterm newborns were six times more likely to develop NH than term newborns. The cause lies in the reduced efficiency of the immature liver and gastrointestinal system in metabolizing bilirubin.^33^^,^^34^ Approximately 15% of the newborns in this study, whose EGA was estimated primarily based on LMP, were preterm. Last menstrual period and symphysis fundal height measurement are the most commonly used methods for estimating EGA in rural areas. Both methods have limitations and lower sensitivity and specificity in determining preterm birth than ultrasound technology.^35^ Symphysis fundal height performs better than LMP, and its accuracy improves by taking more frequent measurements^36^^,^^37^ and by using specific algorithms for the calculation (https://intergrowth21.tghn.org).

Also, newborns who lost excessive weight were six times more likely to develop NH. Many factors can contribute to weight loss. It is more often observed among newborns of first-time mothers who might have difficulties in breastfeeding, as insufficient milk/caloric intake and dehydration can increase the enterohepatic circulation of bilirubin.^38^^,^^39^

Bilirubin at birth (a measure of fetal metabolism) was also associated with later development of NH. In this cohort, levels at birth were significantly elevated in newborns with ABO incompatibility. When intrauterine hemolysis occurs (hemolytic disease of the fetus and newborn), the excess of bilirubin resulting from RBC breakdown is removed by the placenta, and after birth, this excess—especially in the presence of other risk factors such as preterm birth—can lead to severe hyperbilirubinemia.^40^ Also, G6PD-deficient subjects had significantly elevated levels of bilirubin at birth, most likely as a result of degradation of RBCs with a shorter life span.^41^

An early “risk score” based on laboratory and clinical findings in the first 24 hours would be useful in identifying neonates at risk of developing NH and to guide both clinical management and parental counseling in cases of early discharge or when postnatal care is inadequate.^42^ The predictive value of bilirubin at birth from capillary blood and especially from UCB has been extensively studied for this purpose^43^^–^^48^ but with mixed results, and it is not currently recommended by UK NICE Guidelines for risk evaluation of later NH. In our cohort, bilirubin levels in cord blood were, on average, lower than in capillary blood and would not have been useful for screening purposes in the study population.

In this cohort of women enrolled at delivery, we were not able to identify significant maternal risk factors for NH. Although maternal malaria is an important risk factor and prevalence in this cohort was high, we were not able to assess how many episodes occurred during pregnancy, which would have affected fetal development. Maternal anemia was also frequent, but was not associated with NH.

The prevalence of NH in this cohort was lower than reported in other African countries with comparable risk factors.^2^ However, prevalence depends on case definition, which varies according to the guidelines.^49^ Some guidelines define cases based on an absolute TSB cutoff level that varies between countries. Other guidelines recommend the use of a “risk zone,” the percentile above the median of a specific TSB nomogram per hour; however, nomograms may differ because they are generated from specific population data, and different percentiles may be used and combined with risk factors.^27^^,^^50^^–^^53^ South Africa’s national guidelines^54^ recommend thresholds based on birth weight for use in primary level health care, which are probably less accurate than EGA-based thresholds but more applicable in most of rural Africa. Furthermore, bilirubin can be measured from blood (which is prone to interlaboratory differences^55^) or transcutaneously (transcutaneous bilirubin [TcB]). The transcutaneous bilirubinometer is a rapid and non-invasive instrument that has shown good agreement with serum bilirubin in some populations,^56^^,^^57^ whereas in others it overestimated serum values, with variations between devices.^58^^,^^59^ Nomograms for TcB are also available, but not all have been properly validated.

The sample size of this study was small and allowed only the identification of risk factors strongly associated with the outcome of interest or highly prevalent in this population. Furthermore, this was a cross-sectional study in which most women were included at the time of delivery, and this did not allow us to collect more accurate maternal information. Data from the ANC service were limited because of the low number of prenatal visits or quality of the available data or because mothers did not attend that specific service.

We found that 60% of the participants had an adequate knowledge of jaundice, its symptoms, and short- and long-term consequences, and most of them (98%) would take their newborn to the hospital in the presence of symptoms. However, despite providing extensive medical counseling, only one in three mothers whose newborn was diagnosed with hyperbilirubinemia agreed to transfer them to the ICU for phototherapy in the absence of visible jaundice or other symptoms. Community-based jaundice awareness programs should focus on prevention, especially in areas/communities where early hospital discharge rates are high.

## CONCLUSION AND FUTURE DIRECTION

Risk factors for NH are common in this semi-rural area of the DRC, but the lack of diagnostic tools and early discharge from the hospital after delivery suggest that its burden is underestimated. New POC devices to improve the diagnosis of NH and of risk factors could help reduce neonatal morbidity. At the same time, the use of phototherapy cots^60^^,^^61^ and tents with filtered sunlight or solar-powered phototherapy^62^^–^^66^ may help overcome some of the barriers to the use of standard phototherapy.

## Supplemental Materials


Supplemental materials
