# Supplementary material for: Prevalence and Risk Factors of Neonatal Hyperbilirubinemia in a Semi-Rural Area of the Democratic Republic of Congo: A Cohort Study
Source: Am J Trop Med Hyg. 2023 Sep 5;109(4):965–74. doi: 10.4269/ajtmh.23-0293 (PMC10551084; doi:10.4269/ajtmh.23-0293)
Supplement: Supplementary file 1 [file tpmd230293.SD1.pdf]

Table S1. Comparison of characteristics in neonates with and without neonatal hyperbilirubinaemia (analyzed population)

Characteristics	Overall	No jaundice	Jaundice	p-value
Enrolled, n	296*	279*	17*	
Male, n (%)	150 (50.7%)	139 (49.8%)	11 (64.7%)	0.23
Twins, n (%)	11 (3.7%)	10 (3.6%)	1 (5.9%)	0.63
Median gestational age at birth (IQR), wks.	39.5 (38.1, 40.9)	39.6 (38.3, 40.9)	37.6 (36.3, 40.0)	0.046
Mean birthweight (SD), g	3117.4 (470.9)	3119.3 (468.1)	3085.3 (529.1)	0.77
Preterm birth, n (%)	40 (13.5%)	34 (12.2%)	6 (35.3%)	0.007
Low birth weight (<2500 grams), n (%)	22 (7.4%)	20 (7.2%)	2 (11.8%)	0.48
Small for Gestational Age (SGA), n (%)	54/289 (18.7%)	51/273 (18.7%)	3/16 (18.8%)	0.99
Large for Gestational Age (LGA), n (%)	34/289 (11.8%)	31/273 (11.4%)	3/16 (18.8%)	0.37
Mean height (SD), cm	49.3 (2.4)	49.3 (2.4)	49.3 (1.6)	1.00
Mean head circumference (SD), cm	34.3 (1.6)	34.3 (1.6)	34.4 (1.9)	0.78
Mean temperature (SD), degrees C	36.2 (0.5)	36.2 (0.5)	36.2 (0.6)	0.92
Apgar score <7 @ 1 minute, n (%)	22 (7.4%)	19 (6.8%)	3 (17.6%)	0.098
Apgar score <7 @ 5 minutes, n (%)	5 (1.7%)	4 (1.4%)	1 (5.9%)	0.17
Apgar score <7 @ 10 minutes, n (%)	0 (0.0%)	0 (0.0%)	0 (0.0%)	
Mean pulse (SD), beats/min	143.3 (9.7)	143.3 (9.8)	143.8 (8.9)	0.84
Mean respiratory rate (SD), breaths/min	54.0 (4.8)	53.9 (4.8)	55.8 (4.6)	0.12
Mean HCT (SD), %	46.1 (5.0)	46.0 (5.0)	48.2 (5.2)	0.072
Mean Hb (SD), g/dL	15.3 (1.6)	15.3 (1.6)	16.1 (1.7)	0.045
Suspected sepsis, n (%)	3 (1.0%)	1 (0.4%)	2 (11.8%)	<0.001
P. falciparum (RDT), n (%)	4 (1.4%)	4 (1.4%)	0 (0.0%)	0.62
P. falciparum (microscopy), n (%)	1 (0.3%)	1 (0.4%)	0 (0.0%)	0.80
ABO incompatibility, n (%)	37/295 (12.5%)	35/278 (12.6%)	2 (11.8%)	0.92
Rhesus incompatibility, n (%)	2 (0.7%)	2 (0.7%)	0 (0.0%)	0.73
Loss of >7% birthweight, n (%)	36/291 (12.4%)	31/274 (11.3%)	5(29.4%)	0.028
G6PD Deficiency by FST, n (%)	10/295 (3.4%)	7/278 (2.5%)	3 (17.6%)	<0.001
G6PD Deficiency RDT, n (%)	6/177 (3.4%)	4/165 (2.4%)	2/12 (16.7%)	0.008
G6PD Deficiency PCR, n (%)				<0.001
Normal (wildtype)	221/282 (78.4%)	214/266 (80.5%)	7/16 (43.8%)	
Heterozygous	40/282 (14.2%)	37/266 (13.9%)	3/16 (18.8%)	
Hemi- or homozygous	21/282 (7.4%)	15/266 (5.6%)	6/16 (37.5%)	

*Total analyzed if not otherwise indicated

Table S2. Comparison of maternal characteristics in neonates with and without neonatal hyperbilirubinaemia (analyzed population)

Characteristics	Overall	No jaundice	Jaundice	p-value
Included, n	293*	276*	17*	
Teenager, n (%)	41/291 (14.1%)	38/274 (13.9%)	3 (17.6%)	0.66
Primigravid, n (%)	80 (27.3%)	74 (26.8%)	6 (35.3%)	0.45
Median gravidity (IQR)	3.0 (1.0, 4.0)	3.0 (1.0, 4.5)	2.0 (1.0, 4.0)	0.59
Previous newborn diagnosed with NH, n (%)	3 (1.0%)	3 (1.1%)	0 (0.0%)	0.67
Infection in previous month, n (%)	37 (12.6%)	34 (12.3%)	3 (17.6%)	0.52
Median maternal age (IQR)	25.5 (20.8, 32.6)	25.7 (20.8, 32.7)	24.9 (20.2, 28.7)	0.64
Mean maternal weight at birth (SD), kg	64.5 (9.8)	64.7 (10.0)	61.2 (5.7)	0.16
Mean maternal height (SD), cm	162.9 (6.3)	162.9 (6.4)	162.2 (4.9)	0.66
Mean HCT (SD), %	33.9 (4.5)	34.0 (4.4)	32.6 (5.4)	0.21
Mean Hb (SD), g/dL	11.3 (1.5)	11.3 (1.5)	10.9 (1.8)	0.24
Anaemia, n (%)				0.27
Normal (11.0+ g/dL)	185 (63.1%)	178 (64.5%)	7 (41.2%)	
Mild (10.0-10.9 g/dL)	62 (21.2%)	56 (20.3%)	6 (35.3%)	
Moderate (7.0-9.9 g/dL)	45 (15.4%)	41 (14.9%)	4 (23.5%)	
Severe (<7.0 g/dL)	1 (0.3%)	1 (0.4%)	0 (0.0%)	
Median body temperature, degrees C	36.5 (36.3, 36.8)	36.6 (36.3, 36.8)	36.5 (36.3, 37.2)	0.23
Suspected maternal sepsis, n (%)	1 (0.3%)	1 (0.4%)	0 (0.0%)	0.80
P. falciparum (RDT), n (%)	51 (17.4%)	47 (17.0%)	4 (23.5%)	0.49
P. falciparum (microscopy), n (%)	50 (17.1%)	46 (16.7%)	4 (23.5%)	0.47
Geo. mean parasitaemia/µL (95% CI)	1463(708, 3025)	1527(701, 3325)	901(52, 15487)	0.70
Parasitaemia range	32-195000	32-195000	112-7500	
Sickle Cell Trait, n (%)	58/290 (20.0%)	55/273 (20.1%)	3 (17.6%)	0.80
Sickle Cell Disease, n (%)	1/290 (0.3%)	1/273 (0.4%)	0 (0.0%)	0.80
G6PD Deficiency RDT, n (%)	5/179 (2.8%)	4/167 (2.4%)	1/12 (8.3%)	0.23
G6PD Deficiency FST, n (%)	7/291 (2.4%)	5/274 (1.8%)	2 (11.8%)	0.009

*Total analyzed if not otherwise indicated

Table S3. Knowledge & practice of neonatal hyperbilirubinaemia in expecting mothers

Questions	Yes % (n)	No % (n)	Not Known % (n)
**Correctly identified a jaundiced baby in a picture**	59.6 (211)	40.4 (143)	NA
**Have you heard about yellow babies?**	65.5 (232)	34.5 (122)	NA
If yes, from whom (multiple choice):			
Midwife/nurse/Community Health Worker	84.5 (196)	NA	NA
Family/friend/community	13.8 (32)	NA	NA
Traditional birth attendant	0.9 (2)	NA	NA
Personal experience	0.9 (2)	NA	NA
**Do you know why a baby can become yellow?**	11.9 (42)	88.1 (312)	NA
If yes (multiple choice):			
Infection or illness of the baby	56.6 (35)	NA	NA
Infection or illness of the mother	25.9 (14)	NA	NA
Prematurity	3.7 (2)	NA	NA
Drugs taken while pregnant	3.7 (2)	NA	NA
Eat palm oil, groundnut, or other foods while pregnant	1.9 (1)	NA	NA
**Where any of your baby admitted to the hospital because they were yellow?**	1.1 (4)	98.9 (350)	-
If yes (multiple choice):			
Phototherapy	0	NA	NA
Sunlight exposure	(1)	NA	NA
Sweetened water	(1)	NA	NA
Oral traditional treatment	(1)	NA	NA
Drugs (Unspecified)	(1)	NA	NA
**Do you know the symptoms of a baby with jaundice?**	59.9 (212)	41.1 (142)	
If yes (multiple choice):			
Yellow eyes	46.2 (175)	NA	NA
Yellow skin	43.5 (165)	NA	NA
Passing deep yellow urine	8.7 (33)	NA	NA
Other (answers: yellow lips, palm, soles, teethes, fever)	1.6 (6)	NA	NA
**Do you think the yellow skin colour in babies can be harmful?**	59.3 (210)	40.7 (144)	NA
**What can happen if the baby becomes very yellow? *(multiple choice)***			
Baby refuse to feed	47.2 (167)	5.6 (20)	47.2 (167)
Baby becomes sick	69.8 (247)	2.3 (8)	28.0 (99)
Baby's brain is damaged	29.7 (105)	4.0 (14)	66.4 (235)
Baby convulsions	21.2 (75)	3.7 (13)	75.0 (266)
Baby dies	61.3 (217)	2.3 (8)	36.4 (129)
Nothing	0.8 (3)	66.7 (236)	32.5 (115)
**What are the possible consequences on the baby if jaundice is not diagnosed or treated? *(multiple***			

choice)			
Brain damage	29.1 (103)	4.5 (16)	66.4 (235)
Delayed development	30.5 (108)	3.4 (12)	66.1 (234)
Deafness	10.5 (37)	3.7 (13)	85.9 (304)
Death	58.5 (207)	2.3 (8)	39.3 (139)
Convulsions	21.8 (77)	3.1 (11)	75.1 (266)
Other ('baby becomes seriously ill')	(1)	-	-
No effect	0.9 (3)	62.4 (221)	36.7 (130)
**Your baby is 3 days old and his/her skin is yellow, what will you do? *(multiple choice)***			
Bring him/her to the clinic	98.6 (349)	0.3 (1)	1.1 (4)
Give oral herbal/traditional medicine	3.7 (13)	92.9 (329)	3.4 (12)
Apply herbal/traditional medicine on skin	3.1 (11)	93.8 (332)	3.1 (11)
Give extra milk	1.4 (5)	91.2 (323)	7.3 (26)
Sun exposure	3.1 (11)	90.4 (320)	6.5 (23)
Nothing	0.3 (1)	98.3 (348)	1.4 (5)
**If yes to b) or c), what traditional medicine do you (or other people) use when a baby is yellow?**			
Lemon grass/sweet water/can sugar	(4)	-	-
Name of traditional remedy not known	(8)	-	-
**Do you use mothballs in your house? *(multiple choice)***	11.0 (39)	89.0 (315)	-
Room corner, wardrobe, washroom, toilet, roof	84.6 (33)	-	-
Clothes	15.4 (6)	-	-
**Do you use mothballs to store baby cloths/blankets?**	4.8 (17)	95.2 (337)	-
**Do you use other menthol/camphor containing ointments on baby's skin?**	2.3 (8)	97.7 (346)	-
